# The effectiveness of shockwave therapy on patellar tendinopathy, Achilles tendinopathy, and plantar fasciitis: a systematic review and meta-analysis

**DOI:** 10.3389/fimmu.2023.1193835

**Published:** 2023-08-16

**Authors:** Ravon Charles, Lei Fang, Ranran Zhu, Jinxiang Wang

**Affiliations:** ^1^ School of Rehabilitation Science, Shanghai University of Traditional Chinese Medicine, Shanghai, China; ^2^ Engineering Research Centre of Traditional Chinese Medicine Intelligent Rehabilitation, Ministry of Education, Shanghai, China; ^3^ Institute of Rehabilitation Medicine, Shanghai Academy of Traditional Chinese Medicine, Shanghai, China

**Keywords:** extracorporeal shockwave therapy, patellar tendinopathy, Achilles tendinopathy, tendinopathy, plantar fasciitis

## Abstract

**Background:**

Tendinopathy is a growing global concern affecting many people, like athletes, workers, and the elderly. Despite its commonality among the sporting population, there is no practical clinical guideline for patellar tendinopathy (PT). Furthermore, there is conflicting evidence between clinical guidelines on shockwave therapy’s application and clinical utility for Achilles tendinopathy (AT) and plantar fasciitis (PF). Thus, our aim of this study is to evaluate the evidence for shockwave therapy; to provide a Grading of Recommendation, Assessment, Development and Evaluation (GRADE) level of the evidence and effectiveness of shockwave therapy for patellar tendinopathy, Achilles tendinopathy, and Plantar fasciitis.

**Method:**

Medical Literature Analysis and Retrieval System Online (Medline), Embase, The Cumulative Index to Nursing and Allied Health Literature (CINAHL), Physiotherapy Evidence Database (PEDro) and China National Knowledge Infrastructure database (CNKI) were searched to find relevant studies published before December 14^th^, 2022.

**Results:**

Our study showed that for PT in the short term, extracorporeal shockwave therapy (ESWT) or ESWT + eccentric exercise (EE) has a negligible effect on pain and function compared to a placebo or placebo + EE. On the contrary, ESWT significantly affects pain compared to conservative treatment (CT). For AT, ESWT has a small inconclusive effect on pain and function in the short term compared to EE. On the other hand, a placebo outperformed ESWT in improving function for AT but not pain outcomes. PF showed that ESWT significantly affects short- and long-term pain and function. When ESWT was compared to other interventions such as low laser therapy (LLLT), corticosteroid injection (CSI), or CT, there was a small inconclusive effect on pain and function in the short term.

**Conclusion:**

There is low-moderate evidence that ESWT has a negligible effect on pain and function for PT and AT. However, high-quality evidence suggests ESWT has a large effect on pain and function for PF.

**Systematic review registration:**

https://www.crd.york.ac.uk/prospero/display_record.php?ID=CRD42023396835, identifier CRD42023396835.

## 1 Introduction

Tendinopathy is a growing global concern affecting a broad spectrum of people, like athletes, workers, and the elderly (1). Surprisingly, there have been no studies to quantify the total burden of disease that tendinopathy can place on society, governments, and patients (1). Lower limb Tendinopathy (LLT) frequently occurs at the lateral to posterior hip as gluteal tendinopathy, anterior knee as patellar tendinopathy, posteriorly medial ankle as Achilles tendinopathy, or posterior tibialis tendinopathy (2, 3). According to Hopkins et al. (1), characteristics such as sex, age, occupation, physical activity level, and type of sports involved are risk factors for developing LLT. LLT increases with age and affects females more than males (3). The higher rates in females are hypothesized to be due to the metabolic changes that occur during menopause. However, studies have shown that patellar tendinopathy affects athletic men more than athletic women (4, 5).

Patellar tendinopathy (PT) is persistent patellar tendon pain and loss of function related to mechanical loading due to high-impact loading on the knee extensor during physical activities (5, 6). King et al. (4) depicted a prevalence of PT in the athletic population to account for 14.2% of the overall sports injury. Citing sports that demand a high load on the knee extensor complex had a high prevalence, while sports with a low impact on the knee extensor complex had a low prevalence. In elite male soccer/football, approximately 2.4% of players every season get affected by PT, with 61% of these athletes missing up to one week of competition or training session (7). Evidence suggests that Ultrasonographic abnormality is three times as common as clinical symptoms; hence clinical diagnosis is the preferred option for diagnosing PT (8).

Additionally, Achilles tendinopathy (AT) is persistent Achilles tendon pain and loss of function related to mechanical loading due to excessive loading on the plantar flexor complex (9, 10). AT can occur at the midportion of the Achilles tendon or the insertion of the Achilles tendon. AT can affect both the sporting population and the general population. De Jonge et al. (11) showed that the AT incidence rate is 2.53 per 1000 persons in the adult population. Of this, 35% of the cases are related to sporting activities. Although there is a higher cumulative incidence of AT before age 45, the lifetime cumulative incidence of AT is 52% for former endurance runners (12).

Moreover, Plantar fasciitis (PF) is posterior foot pain or heel pain that occurs along the calcaneus to the digits of the foot (13). The prevalence of PF in the general population is approximately 3.6% to 7%; however, PF accounts for 8% of all running-related injuries (14–16). A high body mass index and limited dorsiflexion are the most common risk factors for developing PF (17). As per the conditions mentioned above, PF is also a clinical diagnosis. The first-step pain and pain during weight-bearing activities are the main symptoms (18, 19).

Despite its commonality among the sporting population, there is no practical clinical guideline for patellar tendinopathy. The last systematic review looking at the effectiveness of shockwave therapy for PT was published more than five years ago, and therefore, new trials could have been done in the interim (20, 21). The clinical uncertainty surrounding the level of evidence for shockwave therapy use for PT persists. Furthermore, there needs to be more clarity between clinical guidelines for Achilles tendinopathy, with one clinical guideline that does not recommend shockwave therapy. In contrast, the other clinical guideline does recommend it as an adjunct modality (10, 22). This uncertainty between clinical guidelines could lead to ambiguity among clinicians on whether or not to use shockwave therapy in their clinical practice. Additionally, the latest review that looked at the effectiveness of shockwave therapy for AT used a fixed effect model in their analysis, which does not account for variations in participant characteristics between studies, the distribution of effect sizes, and differences in treatment protocols, dosages, and procedures (23). A similar situation exists in PF clinical guidelines where one guideline recommends it as a secondary approach if standard care fails while the other does not. Apart from this, an updated systematic review is needed due to the previous review investigating the effectiveness of shockwave therapy for PF being ≥ five years (24, 25).

This systematic review aimed to evaluate the evidence for shockwave therapy; to provide a Grading of Recommendations, Assessment, Development and Evaluations (GRADE) level of the evidence and effectiveness of shockwave therapy for patellar tendinopathy, Achilles tendinopathy, and Plantar fasciitis.

## 2 Methods

### 2.1 Search strategy and eligibility criteria

This systematic review was reported according to the Preferred Reporting Items for Systematic Reviews and Meta-Analyses (PRISMA) statement (26). This study did not require ethical approval since the data was obtained exclusively from previously published sources.

An electronic literature search was conducted via four English language databases and one Chinese database; these search databases are as follows: Medical Literature Analysis and Retrieval System Online (Medline) was searched on December 14^th^, 2022, Embase on December 15^th^, 2022, The Cumulative Index to Nursing and Allied Health Literature (CINAHL) on December 18^th^, 2022, Physiotherapy Evidence Database (PEDro) on December 17^th^, 2022 and China National Knowledge Infrastructure database (CNKI) on December 18^th^, 2022. The detailed search strategy is presented in the **Supplementary Material**. The following Boolean operators were used in Medline, Embase, and CINAHL, databases to search for patellar tendinopathy; (patellar tendinopathy) OR (jumper’s knee) OR (patellar tendinosis) OR (patellar tendinitis) OR (patellar tendonitis) AND (shockwave) OR (shockwave therapy) OR (radial shockwave) OR (focused shockwave) OR (extracorporeal shockwave) OR (ESWT). The Boolean operators for Achilles tendinopathy; (Achilles tendinopathy) OR (Mid-portion Achilles tendinopathy) OR (Insertional Achilles tendinopathy) AND (shockwave) OR (shockwave therapy) OR (radial shockwave) OR (focused shockwave) OR (extracorporeal shockwave) OR (ESWT). The Boolean operators for Plantar fasciitis (plantar fasciitis) OR (plantar fasciopathy) OR (heel pain) AND (shockwave) OR (shockwave therapy) OR (radial shockwave) OR (focused shockwave) OR (extracorporeal shockwave) OR (ESWT). Previous systematic reviews were screened for articles and checked citations through google scholar.

The inclusion criteria were: P; People between the ages of 18 or 70 with a clinical diagnosis of patellar tendinopathy, Achilles tendinopathy, and plantar fasciitis of any duration and severity with or without radiological confirmation. I; Studies that included shockwave therapy: radial shockwave therapy or focused shockwave therapy as the mode of treatment. C; Studies compared shockwave therapy to placebo, eccentric exercise, or other interventions. O; Studies that had pain and function as outcome measures. S; completed randomized controlled trials were included in this study. Studies were excluded from the analysis: non-randomized trials, case reports, observational studies, literature reviews, case series, and public conference abstracts. RCTs protocols, uncompleted and unable to access full-text of RCTs were excluded. Studies with missing information regarding the outcome data and the corresponding author who did not respond when contacted for missing data were included in the systematic review for qualitative analysis but were excluded from the quantitative analysis.

### 2.2 Outcome measure

Primary outcome: One primary outcome was pain intensity, measured by a self-reporting tool such as a visual analog scale (VAS), numeric rating scale (NRS), or an equivalent pain perception scale. The other primary outcome was function measured by a self-reporting tool such as the Victorian Institute of sports assessment-patellar (VISA-P), Victorian Institute of sports assessment-Achilles (VISA-A), Foot Function Index (FFI), American Orthopaedic Foot and Ankle Society (AOFAS) and Roles and Maudsley Score (RMS). The timing of the follow-up grouped outcomes: short-term (≤3 months), mid-term (>3 months- ≤ 6 months), and long-term (≥12 months).

### 2.3 Selection of studies and assessment of quality

Two independent reviewers screened all titles and abstracts identified by the search strategy to pinpoint potentially eligible studies. Then, they independently assessed all full texts, and the RCTs that met the inclusion criteria were included in the systematic review. In a disagreement, the two independent reviewers discussed trying to come to a resolution. If no resolution was found, a third reviewer assessed the situation and decided whether or not the article is included or excluded. A commercial reference management software was used for retrieving studies, screening, eliminating duplicates, and managing references.

The internal validity of the included studies was assessed using the Cochrane Collaboration tool for assessing the risk of bias in randomized trials (27). Five domains from this tool were evaluated: Bias arising from the randomization process, bias due to deviation from intended interventions, bias due to missing outcome data, bias in the measurement of the outcome, and bias in the selection of the reported result. The study was classified as having a ‘low,’ ‘high,’ or ‘unclear’ risk of bias based on the two independent reviewer’s judgment of the subcomponents of the Cochrane risk of bias tool. Discrepancies were resolved through discussions. If no resolution was found, a third reviewer assessed the situation and decide on the risk of bias. The Grading of Recommendation Assessment, Development, and Evaluation (GRADE) tool was used to assess the certainty of the evidence (28). This tool comprises five main domains: risk of bias, inconsistency of results, indirectness of evidence, imprecision, and publication bias, assessed independently by two reviewers and resolved through discussion. If no resolution was found, a third reviewer assessed the situation.

### 2.4 Data collection and analysis

Two independent reviewers extracted the data from included studies that characterize the similarities and differences among participants: sample size, sex, age range, country, and type of population. Furthermore, shockwave therapy intervention data were extracted, such as intensity, frequency of treatment, and total duration of the intervention period. The data was compelled and then stored on commercial Microsoft office software.

The meta-analysis was conducted using Revman 5.4 software. Post-treatment means and SDs were extracted from continuous data, while dichotomous data, such as successful outcomes, were extracted. Where there were missing data, the corresponding author was contacted regarding the missing data. If requested data was not provided, attempts to calculate the SDs from CIs, SEs, or p- values were done according to the Cochrane Handbook for systematic reviews of interventions (29). For reporting continuous data, a standardized mean difference (SMD) was calculated, irrespective of whether the outcomes were similar or different. However, only outcomes of a similar construct, for example, pain intensity: VAS and NRS, were combined. The Effect sizes were 0.20-0.49 as small, 0.50-0.79 as medium, and 0.80 or above as large (30). For reporting dichotomous outcomes, odd ratios were used to represent intervention success, where a positive intervention effect >1. The random effects model with a 95% confidence interval was used, assuming variations in participant characteristics between studies, the distribution of effect sizes, and differences in treatment protocols, dosages, and procedures. An I^2^ test measured heterogeneity; an I^2^ value of 25% was low, 50% was moderate, and 75% was high heterogeneity (31). Suppose an analysis includes ten or more studies, and there is statistical significance for heterogeneity with a high percent of I^2^ (>75%). In that case, the prediction interval was examined to determine the estimated effect size distribution. After determining the seriousness of the heterogeneity, further subgroup analysis of the outliers may be performed if warranted. Additionally, if ten or more studies are included in an analysis, a funnel plot was be created to test for publication bias.

## 3 Results

### 3.1 Result of search

**Figure 1:**
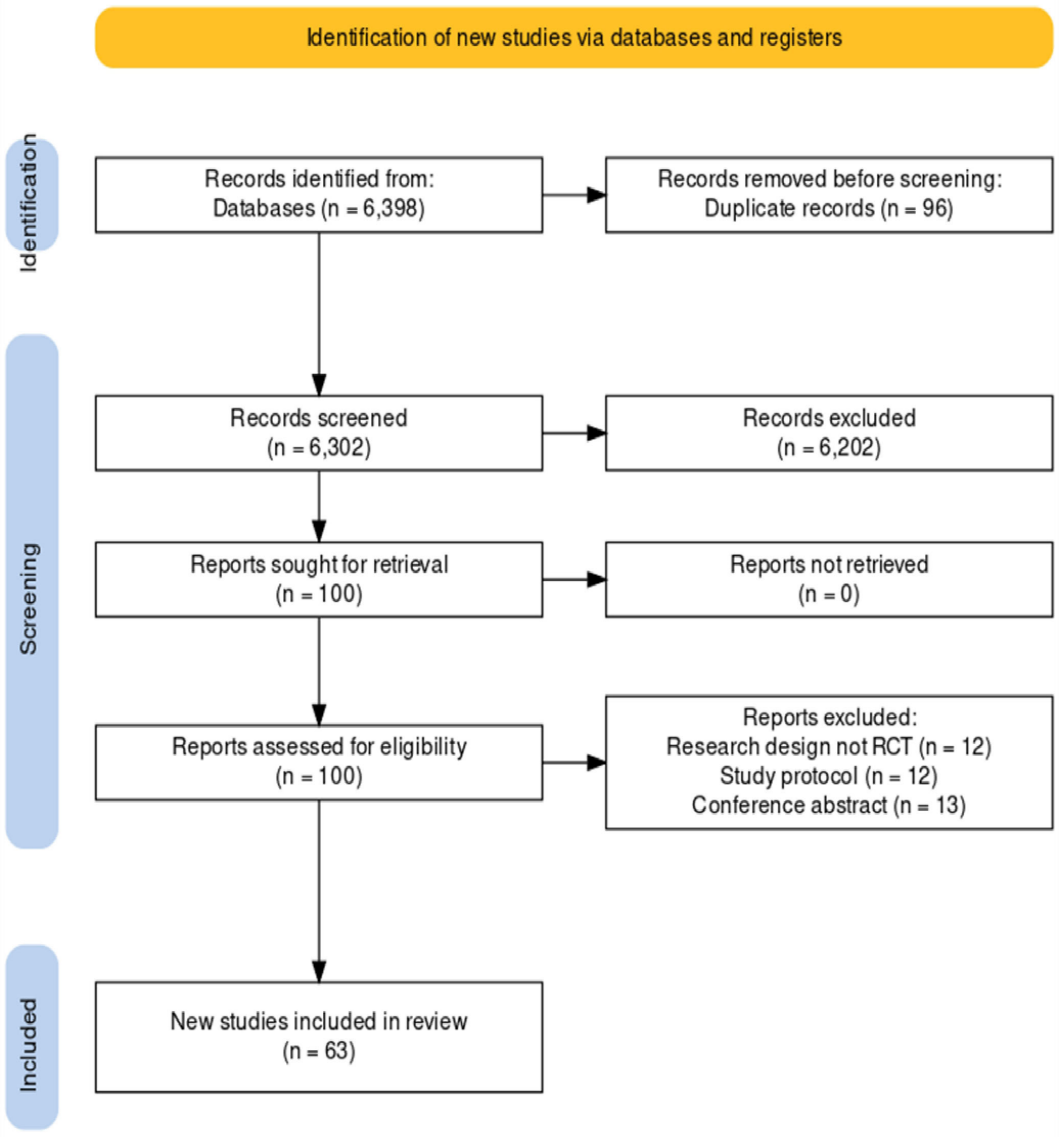
Flowchart of Studies Included.

A total of 6348 articles were identified during the initial search of the above databases and through checking citations on google scholar. After the vigorous screening, sixty-three studies were eligible for this systematic review. **Figure 1** depicts the flow of articles through the search, screening, and inclusion processes.

### 3.2 Characteristics of studies

Sixty-three studies were included in this review; ten studies on patellar tendinopathy, thirteen studies on Achilles tendinopathy, and forty articles on plantar fasciitis. Details about the characteristics of the included studies are shown in **Table 1**.

**Table 1:** Summary of included studies.

Study	Design	Participants	Intervention	Outcome measures
Taunton et al. (2003) (32)	RCT	n =20 age range:23-52; Gender = 10 M, 10 F Population: athlete (not specified)	Exp = ESWT 2000imp level 4 (0.17 mJ/mm^2^) x 3-5TS/3wk – 5wk Con = placebo	• VISA-P • Follow up = 5, 12 wk
Zwerver et al. (2011) (33)	RCT	n = 62 Age (yr) = 24.9 (SD 4.9) Gender = 41 M, 21 F Population: basketball, handball, netball	Exp = ESWT 2000 imp at 4 Hz x 3TS/3wk Con = placebo	• VISA-P • VAS • Follow up = 1, 12, 22wk
Zhang et al. (2020) (34)	RCT	n = 34 Age (yr) = 22.2 (SD 3.8) Gender = 34 M Population: basketball, volleyball	Exp = ESWT 1500imp at 4 Hz Con = placebo	• VISA-P • VAS • Follow up = immediate after treatment
ThØger et al. (2021) (35)	RCT	n = 36 Age (yr) = 31.6 (SD10.4) Gender = 20M, 16F Population: general population	Exp = ESWT 1500-3000imp x 3TS/4wk Con = placebo	• NRS • Follow up = 2, 4, 12wk
Thijs et al. (2017) (36)	RCT	n = 52 Age (yr) = 28.6 (SD6.7) Gender = 38M, 14F Population: physically active	Exp = fESWT+ EE 1000imp at 4 Hz x 3TS/3wk Con = placebo+ EE	• VISA-P • NRS • Follow up = 6, 12, 24wk
Lee et al. (2017) (37)	RCT	n = 34 Gender: 34M Population: volleyball, basketball, handball	Exp = fESWT + EE 1500imp at 4 Hz x 6TS/6wk 3x12 twice per day (5kg) x 12wk Con = placebo + EE	• VISA-P • VAS • Follow up = 12wk
Cheng et al. (2019) (38)	RCT	n= 51 Gender: 25M, 26F Population: judo, volleyball, rowing athlete, basketball, wrestling, weigth-lifting, track and field	Exp = ESWT 2000imp at 9-12 Hz x 16TS/16wk Con = acu + ultrasonic wave therapy	• VAS • Follow up = 16wk
Wang et al. (2007) (39)	RCT	n= 50 Gender: 27M, 23F Population: basketball, jogging, handball, weight lifting, wrestling	Exp = ESWT 1500imp Con = conservative treatment	• VISA-P • VAS • Follow up = 4, 12, 24, 48wk
Van der Worp et al. (2014) (40)	RCT	n= 43 Age (yr) =31.1 (SD10.7) Gender: 32M, 11F Population: sport population	Exp = fESWT + EE 2000imp at 4 Hz x 3TS/3wk Con = rESWT + EE 2000imp at 8Hz x 3TS/3wk	• VISA-P • VAS • Follow up = 7, 14wk
Vetrano et al. (2013) (41)	RCT	n= 46 Gender: 37M, 9F Population: sport population	Exp= ESWT + HE 2000imp x 3TS/1wk Con = PRP + HE	• VISA- • VAS • Follow up = 8, 24, 48wk
Abdelkader et al. (2021) (42)	RCT	n= 50 Gender: 22M, 28F Population: general population	Exp= ESWT + EE 2000imp at 8 Hz x 4TS/4wk 3x15 twice per day x 7d/wk/4wk Con = placebo + EE	• VISA-A • VAS • Follow up = 4, 28, 64wk
Benli et al. (2022) (43)	RCT	n= 60 Age (yr) = 37.3(SD12.2) gender: 23M, 40F Population: sporting population	Exp = ESWT 1500imp at 12Hz Con= EE 3x15 twice per day (5kg which increased 1 kg every two weeks)	• VISA-A • VAS • Follow up = 12, 96wk
Gatz et al. (2021) (44)	RCT	n=66 gender: 40M, 26F Population: general population	Exp= pESWT 2000imp at 5Hz x 4TS/6wk Exp = lESWT 2000imp at 5Hz x 4TS/6wk Con= placebo	• VISA-A • AOFAS • Follow up = 6, 24wk
Vahdatpour et al. (2018) (45)	RCT	n=43 age range: 18-70 gender: 8M, 35F Population: general population	Exp= fESWT 1500imp at 2.3Hz x 4TS/4wk Con= placebo	• AOFAS • VAS • Follow up = 4, 16wk
Lynen et al. (2016) (46)	RCT	n= 59 gender: 28M, 31F Population: general population	Exp = ESWT 1500imp at 4Hz x 3TS/3wk Con = HA	• VISA-A • VAS • Follow up = 4, 12, 24wk
Notarnicola et al. (2013) (47)	RCT	n=60 Population: general population	Exp= ESWT 1600imp x 3TS/2wk Con= CHELT	• VAS • AOFAS • RMS • Follow up = 8, 24wk
Rasmussen et al. (2008) (48)	RCT	n= 48 age range: 18-60 Population: general population	Exp= ESWT 2000imp at 50Hz x 4TS/4wk Con= placebo	• AOFAS • VAS • Follow up = 4, 8, 12wk
Rompe et al. (2006) (49)	RCT	n= 50 age range: 18-70 Population: general population	Exp= ESWT 2000imp at 8Hz x 3TS/3wk Con= EE 3x15 twice per week (5kg) x 7d/wk/12wk Con = WS	• VISA-A • NRS • Follow up = 6, 16wk
Rompe et al. (2008) (50)	RCT	n= 50 gender: 20M, 30F Population: general population	Exp = ESWT 2000imp at 8Hz x 3TS/3wk Con = EE 3x15 twice per week (5kg) x 7d/wk/12wk	• VISA-A • NRS • Follow up = 6, 16wk
Rompe et al. (2009) (51)	RCT	n= 68 gender: 30M, 38F Population: general population	Exp= ESWT + EE 2000imp at 8Hz x 3TS/3wk 3x15 twice per week (5kg) x 7d/wk/12wk Con= EE	• VISA-A • NRS • Follow up = 6, 16wk
Costa et al. (2005) (52)	RCT	n= 49 gender: 21M, 28F Population: general population	Exp= ESWT 1500imp x 3TS/12wk Con= placebo	• VAS • Follow up = 12, 48wk
Mansur et al. (2021) (53)	RCT	n= 119 age range: 18-76 gender: 61M, 58F Population: general population	Exp= rESWT + EE 2000-3000imp at 7-10Hz x 3TS/4wk 3x15 twice per week (5kg) x 7d/wk/12wk Con= EE	• VISA-A • Follow up = 2, 4, 6, 12, 24wk
Pinitkwamdee et al. (2020) (54)	RCT	n= 31 age range= 18-70 gender: 7M, 24F Population: general population	Exp= ESWT 2000imp at 8-12Hz x 4TS/4wk Con = placebo	• VAS • Follow up = 2, 3, 4, 6, 12, 24wk
Alpturker et al. (2020) (55)	RCT	n=40 Age (yr) = 37.78(SD9.86) Gender: 20M, 20F Population: general population	Exp= ESWT 2000imp at 10Hz Con= LLLT	• AOFAS • VAS • RMS • Follow up= 4wk
Asheghan et al. (2019) (56)	RCT	n= 62 age range: 18-75 gender: 20M, 39F Population: general population	Exp= ESWT 2000imp at 10Hz x 3TS/3wk Con= prolotherapy	• VAS • Follow up= 6, 12wk
Bagcier et al. (2020) (57)	RCT	n= 40 Age (yr) = 43.6 (SD 11.8) Gender: 11M, 29F Population: general population	Exp= ESWT 2500imp at 12-15Hz x 3TS/3wk Con= ESWT + DN	• VAS • Follow up= 4wk
Bahar et al. (2020) (58)	RCT	n=45 Age (yr) = 43.6 (SD11.8) Population: general population	Exp = ESWT+ KT 3000imp at 11Hz x 5TS/5wk Con= ESWT + Placebo KT Con = ESWT	• VAS • Follow up= 4wk
Buchbinder et al. (2002) (59)	RCT	n= 166 age range: 18-70 gender: 68M, 93F Population: general population	Exp= ESWT 2000- 2500imp Con= placebo	• VAS • Follow up= 6, 12wk
Caner et al. (2021) (60)	RCT	n= 22 gender: 6M, 16F Population: general population	Exp= ESWT 500imp at 10Hz x 3TS/3wk Con= placebo	• VAS • Follow up= 4, 8wk
Chew et al. (2013) (61)	RCT	n=54 gender: 29M, 25F Population: general population	Exp= ESWT 2000imp x 2TS/2wk Con= ACP Con= CT	• VAS • AOFAS • Follow up= 4, 12, 24wk
Cinar et al. (2018) (62)	RCT	n=66 gender: 10M, 56F Population: general population	Exp= ESWT 2000imp x 3TS/3wk Con= LLLT Con= IEP	• NRS • FFI • Follow up= 3, 12wk
Cinar et al. (2020) (63)	RCT	n= 44 gender: 4M, 40F Population: general population	Exp= ESWT 2000imp x 3TS/3wk Con= IEP	• AOFAS • Follow up= 3, 12wk
Damla et al. (2019) (64)	RCT	n=40 age range: 18-65 Population: general population	Exp= ESWT 2000imp at 10Hz x 3TS/3wk Con= LLLT	• VAS • FFI • Follow up= 4wk
Dingli et al. (2020) (65)	RCT	n= 96 gender: 29M, 68F Population: general population	Exp= ESWT 2000imp x 3TS/3wk Con= CI	• VAS • FFI • Follow up = 12, 24wk
Eslamian et al. (2016) (66)	RCT	n= 40 age range: 18-65 gender: 7M, 33F Population: general population	Exp= ESWT 2000imp x 5TS/2wk Con= CI	• VAS • FFI • Follow up = 4, 8wk
Gerdesmeyer et al. (2008) (67)	RCT	n= 243 Population: general population	Exp= rESWT 2000imp x 3TS/6wk Con= placebo	• VAS • Follow up = 12, 48wk
Gollwitzer et al. (2007) (68)	RCT	n= 40 gender: 15M, 25F Population: general population	Exp= fESWT 2000imp x 3TS/3wk Con= placebo	• VAS • RMS • Follow up = 12wk
Gollwitzer et al. (2015) (69)	RCT	n= 246 Population: general population	Exp= fESWT 2000imp x 3TS/3wk Con= placebo	• VAS • RMS • Follow up= 12wk
Haake et al. (2003) (70)	RCT	n= 271 gender: 67M, F204 Population: general population	Exp= ESWT 4000imp x 3TS/6wk Con= placebo	• VAS • RMS • Follow up = 6, 12, 48wk
Ibrahim et al. (2010) (71)	RCT	n= 50 gender: 18M, 32F Population: general population	Exp= rESWT 2000imp x 2TS/2wk Con= placebo	• VAS • RMS • Follow up = 4, 12, 24wk
Ibrahim et al. (2016) (72)	RCT	n= 50 gender: 18M, 32F Population: general population	Exp= rESWT 2000imp x 2TS/2wk Con= placebo	• VAS • RMS • Follow up = 4, 12, 24, 52wk
Kesikburun et al. (2022) (73)	RCT	n= 27 gender: 7M, 20F Population: general population	Exp= ESWT 1800-2000imp at 4-6Hz x 3TS/6wk Con= prolotherapy	• VAS • RMS • FFI • Follow up = 6, 12wk
Kudo et al. (2006) (74)	RCT	n= 114 gender: 37M, 73F Population: general population	Exp= ESWT 3800imp x 3wk Con= placebo	• VAS • RMS • AOFAS • Follow up = 6w, 12w
Lai et al. (2018) (75)	RCT	n=97 gender: 43M, 54F Population: general population	Exp= ESWT 1500imp x2TS/2wk Con= CI	• VAS • Follow up = 4, 12wk
Malay et al. (2006) (76)	RCT	n= 172 gender: 57M, 115F Population: general population	Exp= ESWT 3800imp Con= placebo	• VAS • Follow up = 4, 8, 12wk
Mardani et al. (2015) (77)	RCT	n= 84 gender: 11M, 57F Population: general population	Exp= ESWT 2000imp x 3TS/3wk Con= CI	• VAS • Follow up = 3, 6, 12wk
Meriç et al. (2018) (78)	RCT	n= 158 gender: 79M, 79F Population: general population	Exp= ESWT 2000imp at 6Hz x 3TS/3wk Con= prolotherapy Con= PRP Con = CI	• VAS • FFI • Follow up = 4, 12, 24, 48, 96, 144wk
Ordahan et al. (2017) (79)	RCT	n=70 gender: 16M, 54F Population: general population	Exp= ESWT 2500imp at 12-15Hz x 5TS/5wk Con= KT	• VAS • Follow up = 5wk
Porter et al. (2005) (80)	RCT	n= 125 gender: 42M, 83F Population: general population	Exp = ESWT 1000imp x 3TS/3wk Con= CI	• VAS • Follow up = 4, 8wk
Rahbar et al. (2018) (81)	RCT	n= 72 gender: 18M, 54F Population: general population	Exp= ESWT 2000imp at 10Hz x 3TS/3wk Con= DN	• VAS • FFI • Follow up= 4, 8wk
Razzano et al. (2017) (82)	RCT	n=104 gender= 53M, 51F Population: general population	Exp= ESWT 2000imp x 3TS/3wk Con= NIN	• VAS • FFI • Follow up = 4, 12wk
Roca et al. (2016) (83)	RCT	n=72 gender: 19M, 53F Population: general population	Exp= ESWT 3000imp at 4Hz Con= BTA	• VAS • RMS • Follow up = 4, 6wk
Rompe et al. (2003) (84)	RCT	n= 45 gender: 22M, 23F Population: general population	Exp= ESWT 2100imp at 4Hz x 3TS/3wk Con= placebo	• VAS • AOFAS • Follow up = 24, 48wk
Rompe et al. (2005) (85)	RCT	n=86 gender: 35M, 51F Population: general population	Exp= ESWT 2000imp x3TS/3wk Con= ESWT + LA	• NRS • AOFAS • Follow up= 3, 12, 48wk
Rompe et al. (2010) (86)	RCT	n= 102 gender: 36M, 66F Population: general population	Exp= rESWT 2000imp at 8Hz x 3TS/3wk Con= PFSS	• FFI • Follow up= 8, 16, 60wk
Rompe et al. (2015) (87)	RCT	n= 152 gender: 71M, 81F Population: general population	Exp= ESWT + PFSS 2000imp at 8Hz x3TS/3wk Con= ESWT	• FFI • Follow up= 8, 16, 96wk
Sibel et al. (2019) (88)	RCT	n=83 gender: 15M, 68F Population: general population	Exp= ESWT 2000imp at 12Hz x 3TS/3wk Con= CFO	• VAS • FFI • Follow up= 4, 12, 24, 48wk
Speed et al. (2003) (89)	RCT	n= 88 gender: 37M, 51F Population: general population	Exp= ESWT 1500imp x 3TS/12wk Con=placebo	• VAS • Follow up= 4, 12wk
Tezel et al. (2020) (90)	RCT	n= 78 gender: 14M, 65F Population: general population	Exp= ESWT 2000imp at 6Hz x 6TS/6wk Con= KT	• VAS • FFI • Follow up= 6wk
Theodore et al. (2004) (91)	RCT	n=150 gender: 41M, 109F Population: general population	Exp= ESWT 1300imp x 3TS/3wk Con= placebo	• RMS • VAS • AOFAS • Follow up= 6, 12, 24, 48wk
Timurtaş et al. (2022) (92)	RCT	n= 47 gender: 9M, 38F Population: general population	Exp= ESWT 1000imp x 3TS/3wk Con= LLLT	• FFI • VAS • Follow up= 3wk
Ulusoy et al. (2017) (93)	RCT	n=60 gender: 12M, 49F Population: general population	Exp= ESWT 2000imp x 3TS/3wk Con= LLLT Con= US	• VAS • AOFAS • RMS • Follow up=4wk
Yucel et al. (2010) (94)	RCT	n= 60 gender: 18M, 42F Population: general population	Exp= ESWT 3000imp Con=CI	• VAS • Follow up= 12wk

Exp, experimental group; Con, control group; ESWT, Extracorporeal shockwave therapy; VISA-P, Victorian Institute of Sports Assessment- Patellar; wk, Week; d/wk, days per week; VAS, Visual Analog Scale S; NRS, Numeric rating scale; fESWT, Focus Extracorporeal shockwave therapy; EE, Eccentric exercise; rESWT, Radial Extracorporeal shockwave therapy; PRP, Platelet-Rich Plasma injection; CT, Conservative treatment; lESWT, Line-focused Extracorporeal shockwave therapy; pESWT, Point-focused Extracorporeal shockwave therapy; AOFAS, American Orthopaedic Foot and Ankle Society; VISA-A, Victorian Institute of Sports Assessment–Achilles; HA, Hyaluronan injections; CHELT, Cold air and High Energy Laser Therapy; RMS, Roles and Maudsley Score; WS, Wait and See; LLLT, Low-level laser therapy; FFI, Foot Function Index; DN, Dye-Needling; KT, Kinesiology Tape; ACP, Autologous Conditioned Plasma; IEP, Insole and exercise program; CI, Corticosteroid Injection; NIN, Noninvasive Interactive Neurostimulation; PFSS, Plantar Fascia-Specific Stretching; CFO, Custom Foot Orthotics; imp, impulse; TS, treatment session; n, sample size; LA, local anesthesia; BTA, botulinum toxin type A; acu, acupuncture.

### 3.3 Risk of bias and certainty of the evidence assessment

**Figure 2:**
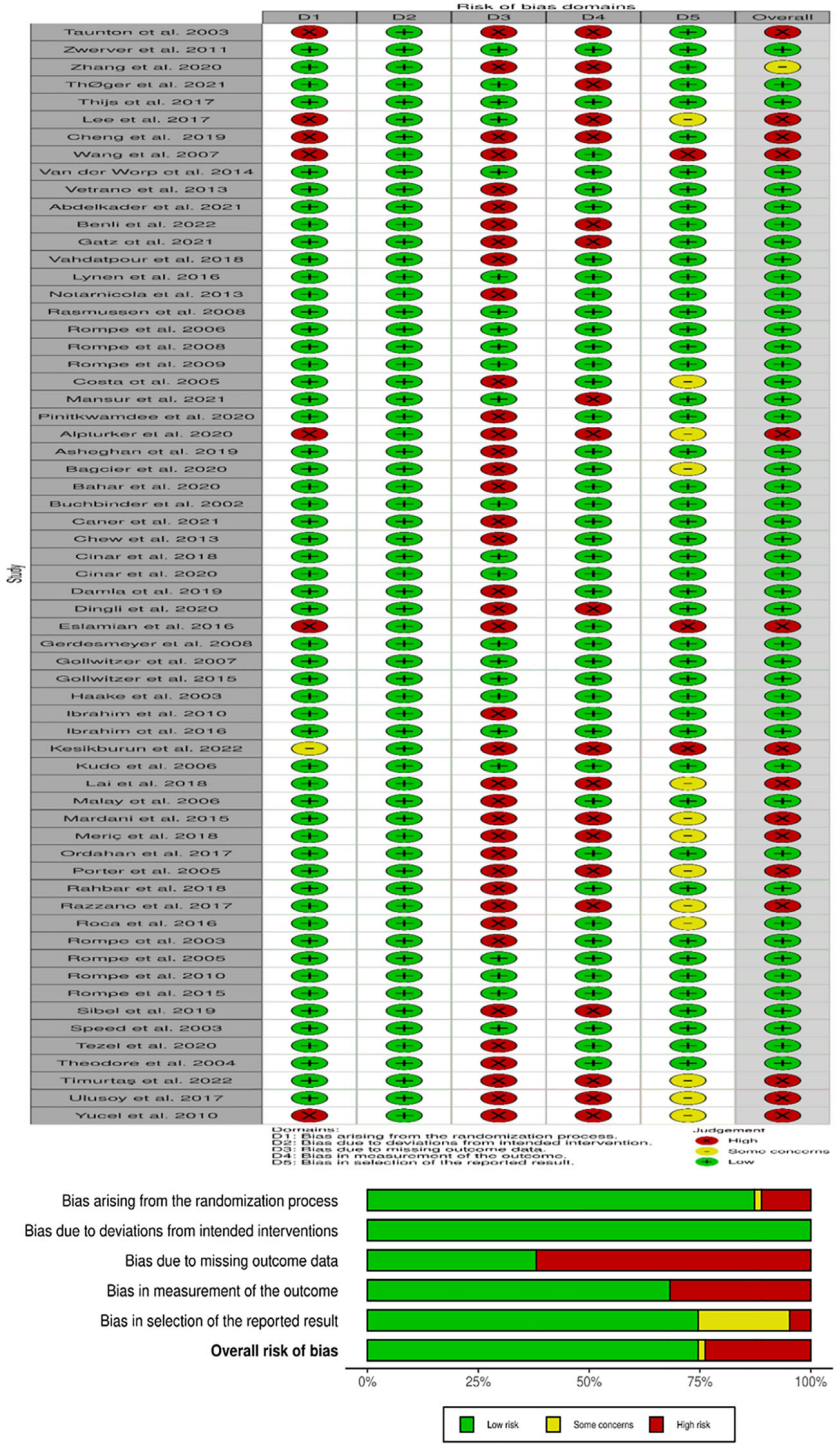
Risk of bias assessment of included studies.

Of the sixty-three studies included in this systematic review, the randomization process was classified as low risk of bias in fifty-five studies, high risk of bias in seven, and with some concerns of bias in one study. All Sixty-three studies had a low risk of bias for deviation from the intended intervention. Missing outcome data had twenty-four studies at low risk and thirty-nine studies at high risk. The risk of bias due to measurement was classified as low in forty-two studies and high in twenty-one studies. The risk of bias in the selection of the reported result was forty-seven articles low, three articles high, and thirteen articles with some concerns of bias. The risk of bias in the included studies is depicted below in **Figure 2**. The detailed GRADE quality assessment result is shown in **Figures 7 to 16 on Supplementary Material**.

### 3.4 Clinical heterogeneity

Nine out of the possible ten studies for patellar tendinopathy were done in the sporting population from varying sports such as basketball, handball, volleyball, football, and track and field. Only one study was done on the general population. In addition, all thirteen included studies looking at Achilles tendinopathy were in the general population. These studies recruited a higher number of males in studies. On the contrary, all forty studies for plantar fasciitis recruited more females than males. Also, the studies were done on the general population.

Four studies compared Extracorporeal Shockwave therapy (ESWT) to placebo only for PT. These studies had very similar shockwave impulses at 1500-3000, the frequency at 4Hz, the treatment session at three weeks, and the duration at 3-5 weeks. The two studies that compared focused ESWT + Eccentric exercise (EE) to placebo + EE showed differences in treatment sessions and durations for shockwave where one study had three treatment sessions over three weeks while the other had six treatment sessions over six weeks; however, they both had similar repetition and set ranges; however different load (intensity). A study compared focused ESWT + EE to radial ESWT + EE, and another study compared radial ESWT against conservative treatment; however, Wang et al. compared ESWT to conservative treatment. The final article for PT compared platelet-rich plasma against ESWT.

Additionally, the six articles that compared ESWT to a placebo for Achilles tendinopathy had vast variations in the treatment session, treatment duration, shockwave impulse, and frequency. Another six studies compared ESWT to EE with identical exercise prescriptions but differences in ESWT protocol. The final two trials compared ESWT to Hyaluronan injection, Cold air, and High Energy Laser Therapy.

Also, thirteen studies compared ESWT to placebo for plantar fasciitis with varying discrepancies in ESWT procedure and protocol. The seven studies that compared ESWT to Corticosteroid injection (CSI) also had variations in ESWT procedure and protocol. In addition, five articles compared ESWT to low laser therapy (LLLT), and three compared ESWT to prolotherapy with a difference in the protocol for the intervention and control groups. The remaining twelve studies that compared ESWT to conservative treatment CT depicted similar inconsistencies. Important to note that the follow-up timepoint varied from immediate (directly after the intervention) to 144 weeks after the intervention.

### 3.5 Evidence of effect on patellar tendinopathy

**Figure 3:**
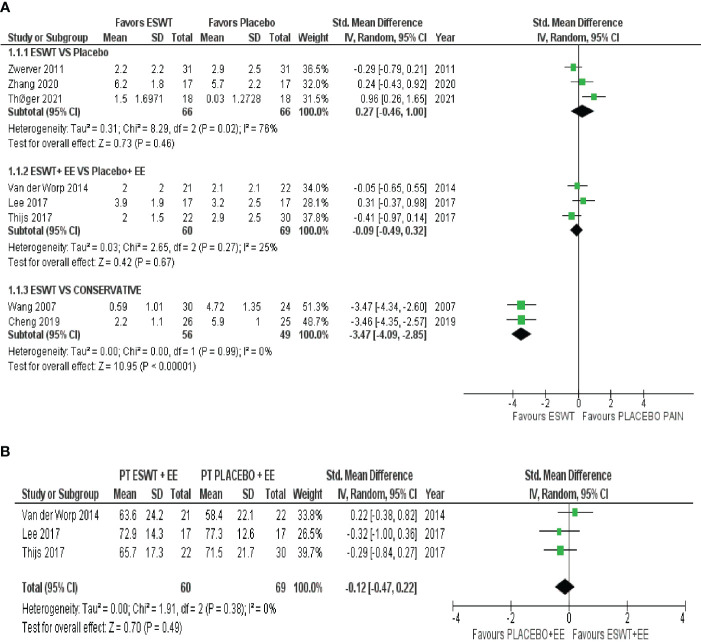
**(A)**: Meta-analysis results and forest plot of the effectiveness of ESWT compared to Placebo, ESWT+ Eccentric exercise versus Placebo+ Eccentric exercise, ESWT compared to Conservative treatment for Patellar tendinopathy pain in the short term. **(B)**: ESWT+ Eccentric exercise versus Placebo+ Eccentric exercise for Patellar tendinopathy function in the short term.

ESWT VS Placebo: based on the analysis of three studies comparing ESWT to placebo, it can be concluded with very low certainty of evidence that ESWT intervention has no superiority over placebo for reducing pain in the short-term. ESWT +EE VS Placebo+ EE: from the analysis of three studies comparing ESWT + EE and placebo + EE, it can be concluded with moderate certainty of evidence that ESWT intervention had a negligible effect in reducing pain but crosses the line of no effect; thus, no conclusive superiority over placebo. Additionally, ESWT had no superiority over placebo for improving function in the short term. ESWT VS Conservative treatment: based on the analysis of two studies comparing ESWT to conservative treatment, it can be concluded with low certainty of evidence that ESWT has a large treatment effect for reducing pain in the short term. A forest plot of the results is presented in **Figures 3A**, **B**.

### 3.6 Evidence of effect on Achilles tendinopathy

**Figure 4:**
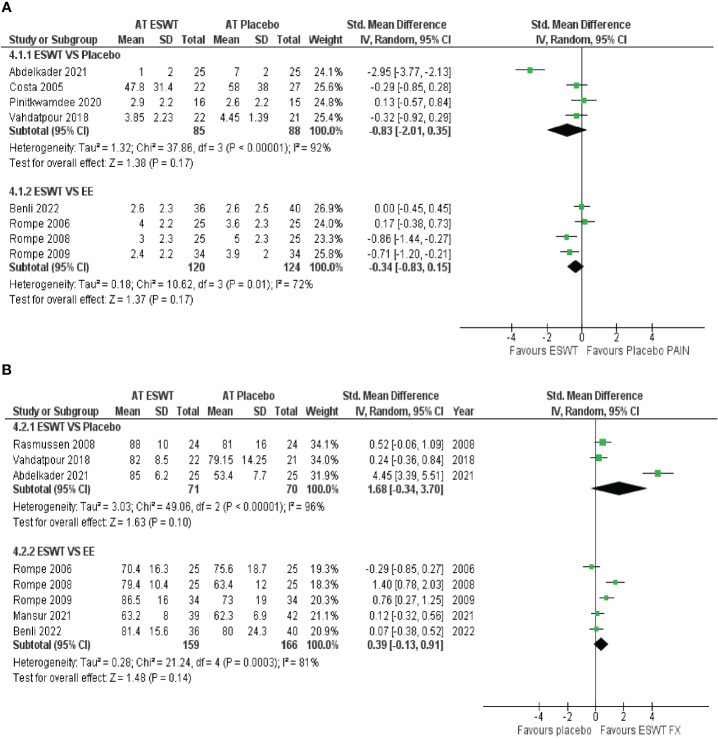
**(A)**: Meta-analysis results and forest plot of the effectiveness of ESWT compared to Placebo, ESWT compared to EE for Achilles tendinopathy for function in the short-term, ESWT compared to Placebo. **(B)**: ESWT compared to EE for Achilles tendinopathy in the short term for pain.

ESWT VS Placebo: From the analysis of four studies comparing ESWT and placebo, it can be concluded with low certainty of evidence that ESWT intervention had a large effect in improving function and reducing pain in the short term but crosses the line of no effect; thus, no conclusive superiority over placebo. ESWT VS EE: Based on the analysis of five studies comparing ESWT to EE, it can be concluded with low certainty of evidence that ESWT intervention had a small effect on reducing pain but crosses the line of no effect; thus, no conclusive superiority over EE. Additionally, ESWT had no superiority over EE for improving function in the short term. A forest plot of the results is presented in **Figures 4A**, **B**.

### 3.7 Evidence of effect on plantar fasciitis

**Figure 5:**
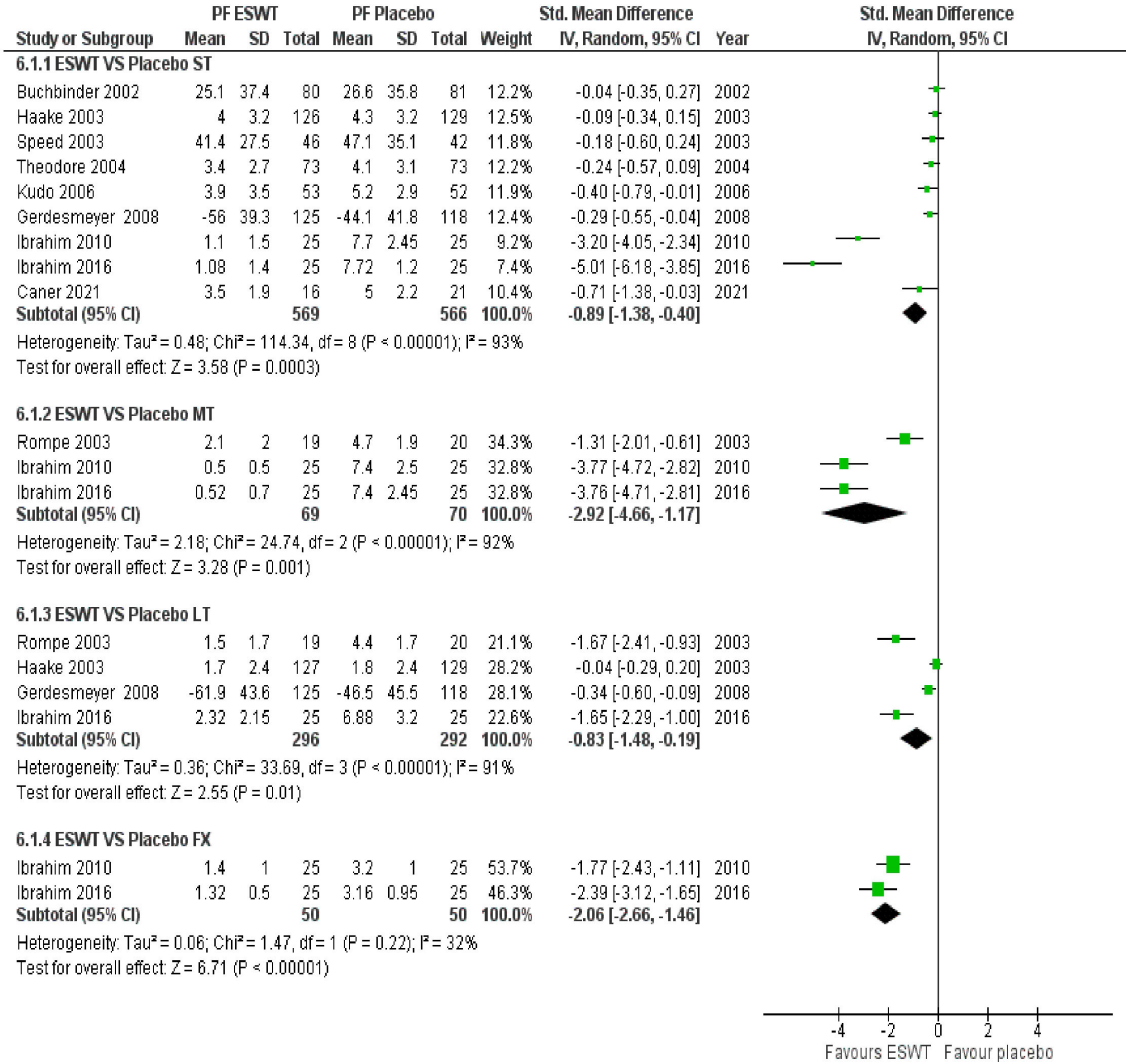
Meta-analysis results and forest plot of the effectiveness of ESWT compared to Placebo for Plantar Fasciitis in the short-term, mid-term and long-term for pain.

**Figure 6:**
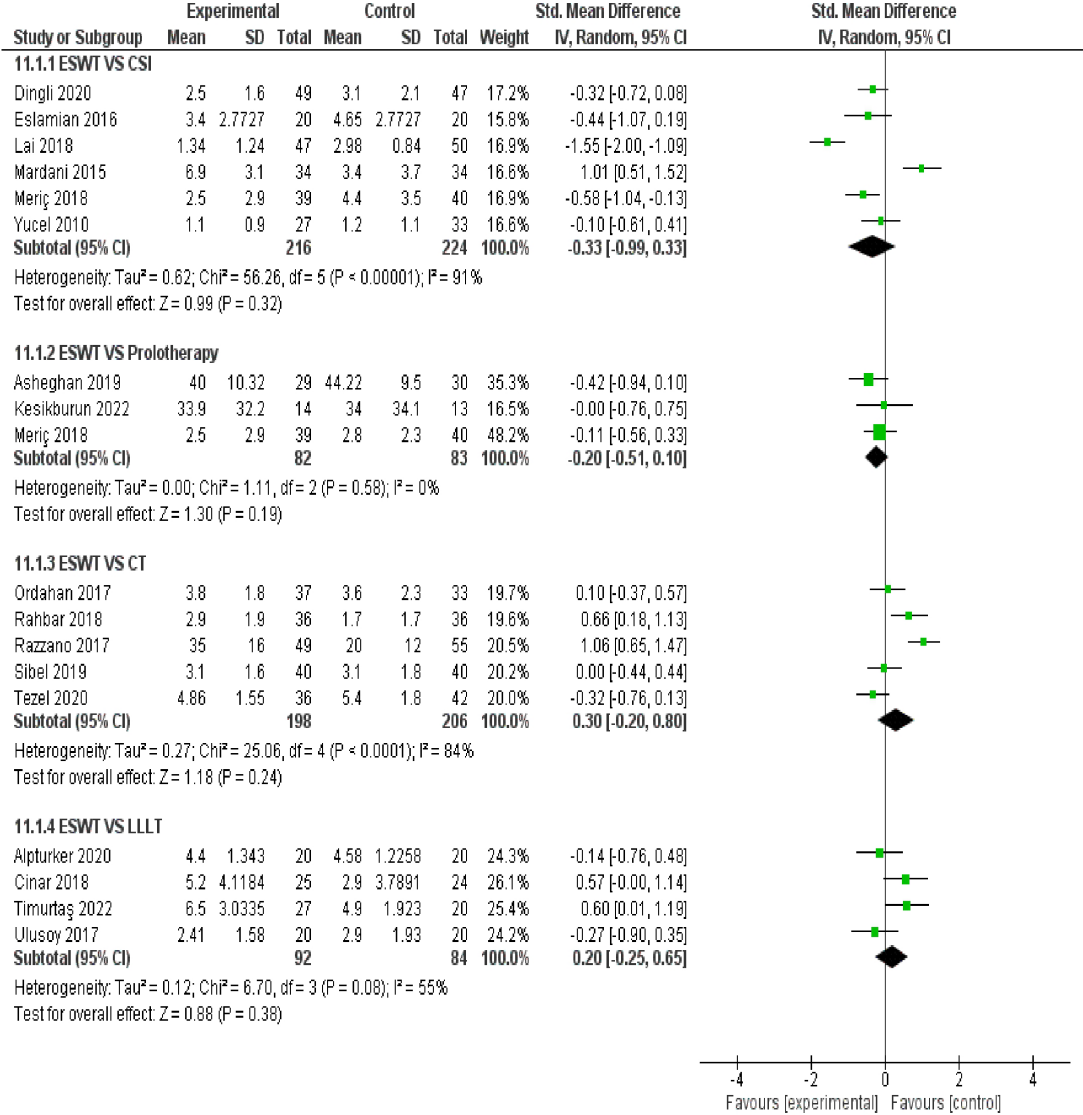
Meta-analysis results and forest plot of the effectiveness of ESWT compared to other treatments for Plantar Fasciitis in the short term for pain.

ESWT VS Placebo: from the analysis of thirteen studies comparing ESWT and placebo, it can be concluded with moderate-high certainty of evidence that ESWT has a large treatment effect for improving function in the short term. Additionally, ESWT has a large treatment effect on reducing pain in the short-term, mid-term, and long-term. ESWT VS CSI: based on the analysis of six studies comparing ESWT to CSI, it can be concluded with very low certainty of evidence that ESWT had a small effect on reducing pain in the short term but crosses the line of no effect thus no conclusive superiority over CSI. ESWT VS LLLT: from the analysis of four studies comparing ESWT and LLLT, it can be concluded with low - moderate certainty of evidence that ESWT intervention had a negligible effect on improving function but crosses the line of no effect; thus, no conclusive superiority over LLLT. Additionally, ESWT had no superiority over LLLT in reducing pain in the short term. ESWT VS Prolotherapy: Based on the analysis of three studies comparing ESWT to Prolotherapy, it can be concluded with low certainty of evidence that ESWT intervention had a small effect on reducing pain in the short term but crosses the line of no effect; thus, no conclusive superiority over prolotherapy. ESWT VS CT: from the analysis of five studies comparing ESWT and CT, it can be concluded with low certainty of evidence that ESWT intervention has no superior over CT for reducing pain in the short term. A forest plot of the results is presented in **Figures 5**, **6**.

### 3.8 Publication bias

No publication bias analysis was done because fewer than ten studies were in an analysis group.

## 4 Discussion

### 4.1 Summary of effects

In this systematic review, we investigated the effectiveness of ESWT on pain and function for patellar tendinopathy, Achilles tendinopathy, and plantar fasciitis compared to a placebo, eccentric exercise, and other interventions. Our study found low to moderate-quality evidence that ESWT or ESWT + EE has a negligible effect on pain and function in the short term compared to a placebo or placebo + EE for PT. On the contrary, we found low-quality evidence that ESWT has a large effect on pain in the short term compared to conservative treatment for PT. For AT, our review found low-quality evidence that ESWT may have a small inconclusive effect on pain and function in the short term compared to EE. On the other hand, a placebo outperformed ESWT in improving function for AT but not pain outcomes. The results for PF showed moderate to high-quality evidence that ESWT has a large effect on pain and function in the short-term, mid-term, and long-term. When ESWT was compared to other interventions such as LLLT, CSI, or CT, there was low to moderate-quality evidence that suggested a small inconclusive effect on pain and function in the short term.

Most studies did not report the type of ESWT used and had vast variations in impulses, frequencies, treatment durations, and sessions. This makes it difficult to comment on an adequate protocol. Some studies did report the type of ESWT, which were Focus Extracorporeal Shockwave Therapy (fESWT) and Radial Extracorporeal Shockwave Therapy (rESWT), respectively; for PF, both fESWT and rESWT had a large effect with high-quality evidence. This implies that both fEWST and rESWT will probably give similar results on pain and function outcomes for PF. Most studies for AT and PT did not report the type of ESWT used. However, in the few studies that reported the type of intervention, negligible effects with low to moderate-quality evidence were shown in both interventions. Additionally, our findings suggest that regardless of the participants from a specific sport or general population, ESWT may have negligible effects with low to moderate quality evidence on the pain and function outcome for PT and AT patients. On the contrary, ESWT likely has a large effect, with high-quality evidence on the pain and function outcome for PF patients from a diverse population.

### 4.2 Summary of previous studies

Due to the lack of clinical practice guidelines for PT, a systematic review of the literature and a GRADE quality assessment of the evidence that outlines the effectiveness of ESWT and ESWT compared to its comparators is imperative to provide clinicians with quality information to make an informed decision about the best available option in managing their patients. Additionally, this review updates the evidence of the previous reviews. Manu et al. (21) conducted a systematic review investigating the effectiveness of ESWT for PT. Their inclusion criteria included both RCTs and non-RCTs, which led to poor methodological studies that may have over or under-estimated the true effect of ESWT for PT. Also, a more recent review by Korakakis et al. (20) had similar shortcomings; hence those reviews likely did not provide a reasonable answer to the effectiveness of ESWT for PT.

Furthermore, ESWT is widely used and recommended by the International Society for Medical Shockwave Treatment (ISMST) (95) for AT; however, prestigious physical therapy practice guideline (22) has recommended against using ESWT for AT leading to uncertainty amongst clinicians on whether ESWT may provide a potential benefit to patients. A recent review (96) seeks to answer the question of ESWT effectiveness for AT, but its methodology needs to be revised. The statistical method used was the fixed model, which does not account for differences in the distribution of effect sizes between studies or participant characteristics and treatment protocols. In addition, the study also included both RCTs and non-RCT trials, which likely led to poor internal validity studies that may have over or under-estimated the actual effect of ESWT for AT; hence an updated review is needed to address these flaws and to provide high-quality evidence.

Additionally, PF faces some similar challenges as AT. Previous guidelines did not recommend ESWT as a form of treatment for PF (18); however, recent guidelines do recommend ESWT for PF (19). The recent review for PF (25) did not provide estimates of the treatment effect magnitude and used a fixed statistical model, which can potentially over or under-estimate the true effect of the intervention (97); hence an updated systematic review quantifying the effectiveness and providing a GRADE quality assessment is essential in addressing this issue.

### 4.3 Implication of results

ESWT is a common modality used in clinical practice to treat musculoskeletal overuse injuries such as tendinopathies and fasciopathies due to its theorized mechanism for healing degenerated soft tissue (2, 19). Our study discovered these novel findings for the effectiveness of ESWT in PT, AT, and PF. ESWT had beneficial short-term effects on pain and function for PT and AT; however, the estimated effects were small and pooled from low to moderate quality evidence, so ESWT should not be used as a primary treatment for modulating pain and function outcomes for PT and AT but as an adjunct in context-specific situations with known treatments that can provide a meaningful change to the patient. On the other hand, ESWT had a large estimated effects on PF in the short to long term, which were pooled from high quality evidence; hence ESWT can be provided as primary treatment for patients with PF.

### 4.4 Recommendations

The majority of the current literature reports on the short-term effectiveness of ESWT for PT, AT, and PF, so future studies with large sample sizes and placebo controls should focus on the efficacy of ESWT in the long term on changing pain and function outcomes in a diverse population. Moreover, future studies should also focus on creating and using a standardized ESWT treatment protocol. In addition, studies evaluating the potential adverse event for ESWT application in PT, AT, and PF are needed.

### 4.5 Limitations

This study had several limitations. First, due to the limited number of included studies in the analysis, the results must be interpreted cautiously because none of our analyses had ten or more studies; hence the results may not accurately represent the true estimated effect for the population (98). Second, Only English and Chinese databases were searched. This could have left out other vital trials relevant to the topic published in other languages. Third, the significance of the heterogeneity observed in the analysis could not be appropriately explained due to the few numbers of included studies.

## 5 Conclusion

In conclusion, there is a negligible effect with a low to moderate certainty of evidence that ESWT can improve function and reduce pain in the short term for PT and AT. However, ESWT had a large effect with a high certainty of evidence in improving function and reducing pain in the short-term, mid-term, and long-term for PF.

## Data availability statement

The original contributions presented in the study are included in the article/**Supplementary Material**. Further inquiries can be directed to the corresponding author.

## Author contributions

RC: protocol design, data collection, extraction, writing, and editing. LF: protocol design, risk of bias assessment, GRADE assessment, data collection, and extraction and reviewing. RZ: risk of bias assessment, data collection and extraction, and GRADE assessment. JW: data interpretation, risk of bias assessment, GRADE assessment, and analysis. All authors contributed to the article and approved the submitted version.
