# Supplementary material for: The effectiveness of shockwave therapy on patellar tendinopathy, Achilles tendinopathy, and plantar fasciitis: a systematic review and meta-analysis
Source: Front Immunol. 2023 Aug 16;14:1193835. doi: 10.3389/fimmu.2023.1193835 (PMC10468604; doi:10.3389/fimmu.2023.1193835)
Supplement: Supplementary file 1 [file DataSheet_1.docx]

Supplementary Material

The Effectiveness of Shockwave Therapy on Patellar Tendinopathy, Achilles Tendinopathy, and Plantar Fasciitis: a systematic review and meta-analysis Ravon Charles, Lei Fang *, Ranran Zhu, Jinxiang Wang

*** Correspondence: Lei Fang: fanglei586@126.com**

# Supplementary Table

**Search Strategy**

**Databases: MEDLINE, EMBASE, CINAHL**

| #1 | (patellar tendinopathy) OR (jumper’s knee) OR (patellar tendinosis) OR (patellar tendinitis) OR (patellar tendonitis) |
| --- | --- |
| #2 | (shockwave) OR (shockwave therapy) OR (radial shockwave) OR (focused shockwave) OR (extracorporeal shockwave) OR (ESWT) |
| #3 | #1 AND #2 |
| #1 | (Achilles tendinopathy) OR (Mid-portion Achilles tendinopathy) OR (Insertional Achilles tendinopathy) |
| #2 | (shockwave) OR (shockwave therapy) OR (radial shockwave) OR (focused shockwave) OR (extracorporeal shockwave) OR (ESWT) |
| #3 | #1 AND #2 |
| #1 | Plantar fasciitis (plantar fasciitis) OR (plantar fasciopathy) OR (heel pain) |
| #2 | (shockwave) OR (shockwave therapy) OR (radial shockwave) OR (focused shockwave) OR (extracorporeal shockwave) OR (ESWT) |
| #3 | #1 AND #2 |

**Table 2. Medline, Embase and CINAHL database search strategy for patellar tendinopathy, Achilles tendinopathy, plantar fasciitis.**

| #1 | bin ji jian yan |
| --- | --- |
| #2 | ti wai cong ji bo |
| #3 | #1 AND #2 |
| #1 | gen jian yan |
| #2 | ti wai cong ji bo |
| #3 | #1 AND #2 |
| #1 | zu di jin mo yan |
| #2 | ti wai cong ji bo |
| #3 | #1 AND #2 |

**Table 3. China National Knowledge Infrastructure database search strategy for patellar tendinopathy, Achilles tendinopathy, plantar fasciitis.**

**Database: PEDro**

Search strategy: Advance

Abstract and title: tendinopathy* *shockwave

Search strategy: Advance

Abstract and title: plantar fasciitis

# Supplementary Figures

| **Shockwave therapy compared to placebo for Patellar Tendinopathy** | | | | | | |
| --- | --- | --- | --- | --- | --- | --- |
| **Patient or population:** Patellar Tendinopathy  **Setting:** outpatient clinic, sports clubs or hospital  **Intervention:** shockwave therapy  **Comparison:** placebo | | | | | | |
| Outcomes | **Anticipated absolute effects^*^** (95% CI) | | Relative effect (95% CI) | № of participants (studies) | Certainty of the evidence (GRADE) | Comments |
|  | **Risk with placebo** | **Risk with shockwave therapy** |  |  |  |  |
| Visual Analog Scale (VAS) | - | SMD **0.27 SD higher** (0.46 lower to 1 higher) | - | 132 (3 RCTs) | ⨁◯◯◯ Very low^a,b,c^ | A very low certainty of evidence that ESWT intervention has no superiority over a placebo in reducing pain |
| ***The risk in the intervention group** (and its 95% confidence interval) is based on the assumed risk in the comparison group and the **relative effect** of the intervention (and its 95% CI).  **CI:** confidence interval; **SMD:** standardised mean difference | | | | | | |
| **GRADE Working Group grades of evidence** **High certainty:** we are very confident that the true effect lies close to that of the estimate of the effect. **Moderate certainty:** we are moderately confident in the effect estimate: the true effect is likely to be close to the estimate of the effect, but there is a possibility that it is substantially different. **Low certainty:** our confidence in the effect estimate is limited: the true effect may be substantially different from the estimate of the effect. **Very low certainty:** we have very little confidence in the effect estimate: the true effect is likely to be substantially different from the estimate of effect. | | | | | | |

**Explanations**

a. not blinding the participants and outcome assessor.

b. A wide dispersion of the estimated effect with a high I2.

c. Wide confidence intervals with the studies crossing the line of no effect.

**Figure 7. GRADE summary of tables for Shockwave therapy compared to placebo for Patellar Tendinopathy.**

|  | | | | | | |
| --- | --- | --- | --- | --- | --- | --- |
| **Shockwave therapy+ eccentric exercise compared to placebo+ eccentric exercise for Patellar Tendinopathy** | | | | | | |
| **Patient or population:** Patellar Tendinopathy  **Setting:** outpatient clinic, sports clubs or hospital  **Intervention:** shockwave therapy+ eccentric exercise  **Comparison:** placebo+ eccentric exercise | | | | | | |
| Outcomes | **Anticipated absolute effects^*^** (95% CI) | | Relative effect (95% CI) | № of participants (studies) | Certainty of the evidence (GRADE) | Comments |
|  | **Risk with placebo+ eccentric exercise** | **Risk with shockwave therapy+ eccentric exercise** |  |  |  |  |
| Victorian Institute of Sports Assessment- Patellar (VISA-P) | - | SMD **0.12 SD lower** (0.47 lower to 0.22 higher) | - | 129 (3 RCTs) | ⨁⨁⨁◯ Moderate^a^ | ESWT had no superiority over a placebo in improving function for PT patients in the short term. |
| Visual Analog Scale (VAS) | - | SMD **0.09 SD lower** (0.49 lower to 0.32 higher) | - | 129 (3 RCTs) | ⨁⨁⨁◯ Moderate^a^ | There is moderate certainty of evidence that ESWT intervention had a negligible effect in reducing pain but crossed the line of no effect; thus, there is no conclusive superiority over the placebo. |
| ***The risk in the intervention group** (and its 95% confidence interval) is based on the assumed risk in the comparison group and the **relative effect** of the intervention (and its 95% CI).  **CI:** confidence interval; **SMD:** standardised mean difference | | | | | | |
| **GRADE Working Group grades of evidence** **High certainty:** we are very confident that the true effect lies close to that of the estimate of the effect. **Moderate certainty:** we are moderately confident in the effect estimate: the true effect is likely to be close to the estimate of the effect, but there is a possibility that it is substantially different. **Low certainty:** our confidence in the effect estimate is limited: the true effect may be substantially different from the estimate of the effect. **Very low certainty:** we have very little confidence in the effect estimate: the true effect is likely to be substantially different from the estimate of effect. | | | | | | |

**Explanations**

a. A wide confidence interval with studies crossing the line of no effect.

**Figure8. GRADE summary of tables for Shockwave therapy+ eccentric exercise compared to placebo+ eccentric exercise for Patellar Tendinopathy.**

|  | | | | | | |
| --- | --- | --- | --- | --- | --- | --- |
| **Shockwave therapy compared to conservative treatment for Patellar Tendinopathy** | | | | | | |
| **Patient or population:** Patellar Tendinopathy  **Setting:** outpatient clinic, sports clubs or hospital  **Intervention:** shockwave therapy  **Comparison:** conservative treatment | | | | | | |
| Outcomes | **Anticipated absolute effects^*^** (95% CI) | | Relative effect (95% CI) | № of participants (studies) | Certainty of the evidence (GRADE) | Comments |
|  | **Risk with conservative treatment** | **Risk with shockwave therapy** |  |  |  |  |
| Visual Analog Scale (VAS) | - | SMD **3.47 SD lower** (4.09 lower to 2.85 lower) | - | 105 (2 RCTs) | ⨁⨁◯◯ Low^a,b^ | There is a low certainty of evidence that ESWT has a large treatment effect in reducing pain for PT patients in the short term. |
| ***The risk in the intervention group** (and its 95% confidence interval) is based on the assumed risk in the comparison group and the **relative effect** of the intervention (and its 95% CI).  **CI:** confidence interval; **SMD:** standardised mean difference | | | | | | |
| **GRADE Working Group grades of evidence** **High certainty:** we are very confident that the true effect lies close to that of the estimate of the effect. **Moderate certainty:** we are moderately confident in the effect estimate: the true effect is likely to be close to the estimate of the effect, but there is a possibility that it is substantially different. **Low certainty:** our confidence in the effect estimate is limited: the true effect may be substantially different from the estimate of the effect. **Very low certainty:** we have very little confidence in the effect estimate: the true effect is likely to be substantially different from the estimate of effect. | | | | | | |

**Explanations**

a. no blinding of the participants, outcome assessor and no allocation concealment.

b. multiple interventions

**Figure9. GRADE summary of tables for Shockwave therapy compared to conservative treatment for Patellar Tendinopathy.**

| **Shockwave therapy compared to placebo for Achilles tendinopathy** | | | | | | |
| --- | --- | --- | --- | --- | --- | --- |
| **Patient or population:** Achilles tendinopathy  **Setting:** outpatient clinic, sports clubs or hospital  **Intervention:** shockwave therapy  **Comparison:** placebo | | | | | | |
| Outcomes | **Anticipated absolute effects^*^** (95% CI) | | Relative effect (95% CI) | № of participants (studies) | Certainty of the evidence (GRADE) | Comments |
|  | **Risk with placebo** | **Risk with shockwave therapy** |  |  |  |  |
| Numeric Rating Scale (NRS) | - | SMD **0.83 SD lower** (2.01 lower to 0.35 higher) | - | 173 (4 RCTs) | ⨁⨁◯◯ Low^a,b^ | There is low certainty of evidence that ESWT intervention had a large effect in reducing pain for AT in the short term but crosses the line of no effect; thus, there is no conclusive superiority over placebo. |
| American Orthopaedic Foot and Ankle Society (AOFAS) | - | SMD **1.68 SD higher** (0.34 lower to 3.7 higher) | - | 141 (3 RCTs) | ⨁⨁◯◯ Low^a,b^ | There is low certainty of evidence that ESWT intervention had a large effect in improving function for AT in the short term but crosses the line of no effect; thus, there is no conclusive superiority over placebo. |
| ***The risk in the intervention group** (and its 95% confidence interval) is based on the assumed risk in the comparison group and the **relative effect** of the intervention (and its 95% CI).  **CI:** confidence interval; **SMD:** standardised mean difference | | | | | | |
| **GRADE Working Group grades of evidence** **High certainty:** we are very confident that the true effect lies close to that of the estimate of the effect. **Moderate certainty:** we are moderately confident in the effect estimate: the true effect is likely to be close to the estimate of the effect, but there is a possibility that it is substantially different. **Low certainty:** our confidence in the effect estimate is limited: the true effect may be substantially different from the estimate of the effect. **Very low certainty:** we have very little confidence in the effect estimate: the true effect is likely to be substantially different from the estimate of effect. | | | | | | |

**Explanations**

a. A wide dispersion of the estimated effect with a high I2.

b. A wide confidence interval with studies crossing the line of no effect.

**Figure10. GRADE summary of tables for Shockwave therapy compared to placebo for Achilles Tendinopathy.**

|  | | | | | | |
| --- | --- | --- | --- | --- | --- | --- |
| **Shockwave therapy compared to eccentric exercise for Achilles tendinopathy** | | | | | | |
| **Patient or population:** Achilles tendinopathy  **Setting:** outpatient clinic, sports clubs or hospital  **Intervention:** shockwave therapy  **Comparison:** eccentric exercise | | | | | | |
| Outcomes | **Anticipated absolute effects^*^** (95% CI) | | Relative effect (95% CI) | № of participants (studies) | Certainty of the evidence (GRADE) | Comments |
|  | **Risk with eccentric exercise** | **Risk with shockwave therapy** |  |  |  |  |
| Victorian Institute of Sports assessment-Achilles (VISA-A) | - | SMD **0.39 SD higher** (0.13 lower to 0.91 higher) | - | 325 (5 RCTs) | ⨁⨁◯◯ Low^a,b^ | ESWT had no superiority over EE in improving function (SMD 0.39, 95% CI -0.13 to 0.91) for AT in the short term. |
| Numeric Rating Scale (NRS) | - | SMD **0.34 SD lower** (0.83 lower to 0.15 higher) | - | 244 (4 RCTs) | ⨁⨁◯◯ Low^a,c^ | There is low certainty of evidence that ESWT intervention had a small effect on reducing pain but crosses the line of no effect; thus, there is no conclusive superiority over EE. |
| ***The risk in the intervention group** (and its 95% confidence interval) is based on the assumed risk in the comparison group and the **relative effect** of the intervention (and its 95% CI).  **CI:** confidence interval; **SMD:** standardised mean difference | | | | | | |
| **GRADE Working Group grades of evidence** **High certainty:** we are very confident that the true effect lies close to that of the estimate of the effect. **Moderate certainty:** we are moderately confident in the effect estimate: the true effect is likely to be close to the estimate of the effect, but there is a possibility that it is substantially different. **Low certainty:** our confidence in the effect estimate is limited: the true effect may be substantially different from the estimate of the effect. **Very low certainty:** we have very little confidence in the effect estimate: the true effect is likely to be substantially different from the estimate of effect. | | | | | | |

**Explanations**

a. A wide dispersion of the estimated effect with a high I2.

b. A wide confidence interval with studies crossing the line of no effect.

c. A wide dispersion of prediction interval with a high I2.

**Figure11. GRADE summary of tables for Shockwave therapy compared to eccentric exercise for Achilles Tendinopathy.**

|  | | | | | | |
| --- | --- | --- | --- | --- | --- | --- |
| **Shockwave therapy compared to placebo for plantar fasciitis** | | | | | | |
| **Patient or population:** plantar fasciitis  **Setting:** outpatient clinic, sports clubs or hospital  **Intervention:** shockwave therapy  **Comparison:** placebo | | | | | | |
| Outcomes | **Anticipated absolute effects^*^** (95% CI) | | Relative effect (95% CI) | № of participants (studies) | Certainty of the evidence (GRADE) | Comments |
|  | **Risk with placebo** | **Risk with shockwave therapy** |  |  |  |  |
| Visual Analogue Scale (VAS) | - | SMD **0.89 SD lower** (1.38 lower to 0.4 lower) | - | 1135 (9 RCTs) | ⨁⨁⨁◯ Moderate^a^ | ESWT has a large treatment effect on reducing pain for PF patients in the short-term, mid-term and long-term for PF |
| Roles and Maudsley score (RMS) | - | SMD **2.06 SD lower** (2.66 lower to 1.46 lower) | - | 100 (2 RCTs) | ⨁⨁⨁⨁ High | There is a high certainty of evidence that ESWT has a large treatment effect in improving function for PF in the short term. |
| Visual Analogue Scale Odds Ratio (VAS) | 323 per 1,000 | **481 per 1,000** (419 to 543) | **OR 1.94** (1.51 to 2.49) | 1146 (7 RCTs) | ⨁⨁⨁⨁ High |  |
| ***The risk in the intervention group** (and its 95% confidence interval) is based on the assumed risk in the comparison group and the **relative effect** of the intervention (and its 95% CI).  **CI:** confidence interval; **OR:** odds ratio; **SMD:** standardised mean difference | | | | | | |
| **GRADE Working Group grades of evidence** **High certainty:** we are very confident that the true effect lies close to that of the estimate of the effect. **Moderate certainty:** we are moderately confident in the effect estimate: the true effect is likely to be close to the estimate of the effect, but there is a possibility that it is substantially different. **Low certainty:** our confidence in the effect estimate is limited: the true effect may be substantially different from the estimate of the effect. **Very low certainty:** we have very little confidence in the effect estimate: the true effect is likely to be substantially different from the estimate of effect. | | | | | | |

**Explanations**

a. A wide dispersion of the estimated effect with a high I2.

**Figure 12. GRADE summary of tables for Shockwave therapy compared to placebo for Plantar Fasciitis.**

|  | | | | | | |
| --- | --- | --- | --- | --- | --- | --- |
| **Shockwave therapy compared to low level laser therapy for plantar fasciitis** | | | | | | |
| **Patient or population:** plantar fasciitis  **Setting:** outpatient clinic, sports clubs or hospital  **Intervention:** shockwave therapy  **Comparison:** low level laser therapy | | | | | | |
| Outcomes | **Anticipated absolute effects^*^** (95% CI) | | Relative effect (95% CI) | № of participants (studies) | Certainty of the evidence (GRADE) | Comments |
|  | **Risk with low level laser therapy** | **Risk with shockwave therapy** |  |  |  |  |
| American Orthopaedic Foot and Ankle Society (AOFAS) | - | SMD **0.13 SD higher** (0.31 lower to 0.57 higher) | - | 80 (2 RCTs) | ⨁⨁◯◯ Low^a,b^ | There is low certainty of evidence that ESWT intervention had a negligible effect on improving function but crossed the line of no effect; thus, no conclusive superiority over LLLT. |
| Visual Analogue Scale (VAS) | - | SMD **0.2 SD higher** (0.25 lower to 0.65 higher) | - | 176 (4 RCTs) | ⨁⨁⨁◯ Moderate^a^ | ESWT had no superiority over LLLT in reducing pain for PF in the short term. |
| ***The risk in the intervention group** (and its 95% confidence interval) is based on the assumed risk in the comparison group and the **relative effect** of the intervention (and its 95% CI).  **CI:** confidence interval; **SMD:** standardised mean difference | | | | | | |
| **GRADE Working Group grades of evidence** **High certainty:** we are very confident that the true effect lies close to that of the estimate of the effect. **Moderate certainty:** we are moderately confident in the effect estimate: the true effect is likely to be close to the estimate of the effect, but there is a possibility that it is substantially different. **Low certainty:** our confidence in the effect estimate is limited: the true effect may be substantially different from the estimate of the effect. **Very low certainty:** we have very little confidence in the effect estimate: the true effect is likely to be substantially different from the estimate of effect. | | | | | | |

**Explanations**

a. Did not blind participant and outcome assessor.

b. A wide confidence interval with studies crossing the line of no effect.

**Figure13. GRADE summary of tables for Shockwave therapy compared to Low-Level Laser therapy for Plantar Fasciitis.**

| **Shockwave therapy compared to Corticosteroid Injection for plantar fasciitis** | | | | | | |
| --- | --- | --- | --- | --- | --- | --- |
| **Patient or population:** plantar fasciitis  **Setting:** outpatient clinic, sports clubs or hospital  **Intervention:** shockwave therapy  **Comparison:** Corticosteroid Injection | | | | | | |
| Outcomes | **Anticipated absolute effects^*^** (95% CI) | | Relative effect (95% CI) | № of participants (studies) | Certainty of the evidence (GRADE) | Comments |
|  | **Risk with Corticosteroid Injection** | **Risk with shockwave therapy** |  |  |  |  |
| Visual analogue scale (VAS) | - | SMD **0.33 SD lower** (0.99 lower to 0.33 higher) | - | 440 (6 RCTs) | ⨁◯◯◯ Very low^a,b,c^ | There is a very low certainty of evidence that ESWT had a small effect on reducing pain for AT in the short term but crossed the line of no effect thus no conclusive superiority over CI. |
| ***The risk in the intervention group** (and its 95% confidence interval) is based on the assumed risk in the comparison group and the **relative effect** of the intervention (and its 95% CI).  **CI:** confidence interval; **SMD:** standardised mean difference | | | | | | |
| **GRADE Working Group grades of evidence** **High certainty:** we are very confident that the true effect lies close to that of the estimate of the effect. **Moderate certainty:** we are moderately confident in the effect estimate: the true effect is likely to be close to the estimate of the effect, but there is a possibility that it is substantially different. **Low certainty:** our confidence in the effect estimate is limited: the true effect may be substantially different from the estimate of the effect. **Very low certainty:** we have very little confidence in the effect estimate: the true effect is likely to be substantially different from the estimate of effect. | | | | | | |

**Explanations**

a. Did not blind participants and outcome assessor.

b. A wide dispersion of the estimated effect with a high I2.

c. A wide confidence interval with studies crossing the line of no effect.

**Figure14. GRADE summary of tables for Shockwave therapy compared Corticosteroid Injection for Plantar Fasciitis.**

|  | | | | | | |
| --- | --- | --- | --- | --- | --- | --- |
| **Shockwave therapy compared to prolotherapy for plantar fasciitis** | | | | | | |
| **Patient or population:** plantar fasciitis  **Setting:** outpatient clinic, sports clubs or hospital  **Intervention:** shockwave therapy  **Comparison:** prolotherapy | | | | | | |
| Outcomes | **Anticipated absolute effects^*^** (95% CI) | | Relative effect (95% CI) | № of participants (studies) | Certainty of the evidence (GRADE) | Comments |
|  | **Risk with prolotherapy** | **Risk with shockwave therapy** |  |  |  |  |
| Visual analogue scale (VAS) | - | SMD **0.2 SD lower** (0.51 lower to 0.1 higher) | - | 165 (3 RCTs) | ⨁⨁◯◯ Low^a,b^ | There is a low certainty of evidence that ESWT intervention had a small effect on reducing pain for PF in the short term but crossed the line of no effect; thus, no conclusive superiority over prolotherapy. |
| ***The risk in the intervention group** (and its 95% confidence interval) is based on the assumed risk in the comparison group and the **relative effect** of the intervention (and its 95% CI).  **CI:** confidence interval; **SMD:** standardised mean difference | | | | | | |
| **GRADE Working Group grades of evidence** **High certainty:** we are very confident that the true effect lies close to that of the estimate of the effect. **Moderate certainty:** we are moderately confident in the effect estimate: the true effect is likely to be close to the estimate of the effect, but there is a possibility that it is substantially different. **Low certainty:** our confidence in the effect estimate is limited: the true effect may be substantially different from the estimate of the effect. **Very low certainty:** we have very little confidence in the effect estimate: the true effect is likely to be substantially different from the estimate of effect. | | | | | | |

**Explanations**

a. Did not blind the participant and outcome assessor.

b. A wide confidence interval with studies crossing the line of no effect.

**Figure15. GRADE summary of tables for Shockwave therapy compared to Prolotherapy for Plantar Fasciitis.**

|  | | | | | | |
| --- | --- | --- | --- | --- | --- | --- |
| **Shockwave therapy compared to conservative treatment for plantar fasciitis** | | | | | | |
| **Patient or population:** plantar fasciitis  **Setting:** outpatient clinic, sports clubs or hospital  **Intervention:** shockwave therapy  **Comparison:** conservative treatment | | | | | | |
| Outcomes | **Anticipated absolute effects^*^** (95% CI) | | Relative effect (95% CI) | № of participants (studies) | Certainty of the evidence (GRADE) | Comments |
|  | **Risk with conservative treatment** | **Risk with shockwave therapy** |  |  |  |  |
| Visual analogue scale (VAS) | - | SMD **0.3 SD higher** (0.2 lower to 0.8 higher) | - | 404 (5 RCTs) | ⨁⨁◯◯ Low^a,b^ | There is low certainty of evidence that ESWT intervention has no superior over CT in reducing pain for PF in the short term. |
| ***The risk in the intervention group** (and its 95% confidence interval) is based on the assumed risk in the comparison group and the **relative effect** of the intervention (and its 95% CI).  **CI:** confidence interval; **SMD:** standardised mean difference | | | | | | |
| **GRADE Working Group grades of evidence** **High certainty:** we are very confident that the true effect lies close to that of the estimate of the effect. **Moderate certainty:** we are moderately confident in the effect estimate: the true effect is likely to be close to the estimate of the effect, but there is a possibility that it is substantially different. **Low certainty:** our confidence in the effect estimate is limited: the true effect may be substantially different from the estimate of the effect. **Very low certainty:** we have very little confidence in the effect estimate: the true effect is likely to be substantially different from the estimate of effect. | | | | | | |

**Explanations**

a. A wide dispersion of the estimated effect with a high I2.

b. A wide confidence interval with studies crossing the line of no effect.

**Figure16. GRADE summary of tables for Shockwave therapy compared to conservative treatment for Plantar Fasciitis.**
